# On the Means of Preventing the Spasmodic Cholera

**Published:** 1831

**Authors:** 


### On the Means of preventing the Spasmodic Cholera.

(Tenth Observation.)*

In a former observation, I have attributed the epidemic form of cholera to a vitiated atmosphere acting on predisposed persons; we have yet to discover the source and nature of this depraved air before we can counteract its influence, and the state of the human body which lays it open to this disease before we can form a code of prophylactic measures.

The occurrence of spasmodic cholera under every variety of weather, and its return at uncertain periods, as well as its partial dispersion by even slight changes in the currents of the atmosphere, prove that the miasm is not generally distributed, and certainly lead to the supposition that animal or vegetable exhalations, where subjected to the action of an atmosphere possessing peculiar properties, form with it the

---

* This additional observation did not arrive in time to be added to Dr. Smith's paper on Cholera, published in the 28th Number of this Journal.

exciting cause of the disease. But cholera makes its attacks in situations so different from each other, that there is often no point of local resemblance; at one time it appears many hundred miles from the sea, at another, on its very shores; sometimes on low marshy grounds, often on dry lands: and narrow strips of country have suffered from its ravages, while both sides of these tracts have been exempt. We cannot, therefore admit that marsh malaria has any thing to do with the production of cholera; and the communication of the disease by infection, in India, is hardly acknowledged, I believe, by any one who has watched its progress in that country. When we bear in mind, too, that cholera varies its aspect, or at least its mode of progress, with every season, we are compelled to hasten to the confession, that nothing but local and immediate investigation, with experimental analysis, can lead to the discovery of the true causes and constitution of the prevailing epidemic, and to the means which are most likely to prevent it.

Of the predisposing causes, or state of body which is obnoxious to spasmodic cholera, there is little more certainly known than of its external or exciting causes. We only know that no age, rank, sex, or character is safe from its attack; and while a malady thus sets at nought every physical law, it can afford but slender grounds for the formation of a prophylactic code. In the present state of our knowledge, perhaps the following plan might be as good as any.

The establishment in every town of a board of health, composed of the magistrates, clergy, and medical men of the place, who would visit all the manufactories and receptacles of the poor and working classes, remove the people from their laborious employments before fatigue or exhaustion were experienced, and ensure to them a sufficiency of wholesome diet, with a due proportion of good beer. The members of these boards ought also to have power to suppress all petty spirit shops, and to enable every poor family to brew a wholesome ale at home. They should lessen, by every possible means, mental anxiety and discontent, and, when disease appeared, exert every faculty to discover its sources and to destroy them.

---

VIII.

